# 0.6 V, 116 nW Neural Spike Acquisition IC with Self-Biased Instrumentation Amplifier and Analog Spike Extraction

**DOI:** 10.3390/s18082460

**Published:** 2018-07-30

**Authors:** Jong Pal Kim, Hankyu Lee, Hyoungho Ko

**Affiliations:** 1 Multimedia Processing Lab., Samsung Advanced Institute of Technology (SAIT), Suwon 16678, Korea; jongpalk@samsung.com (J.P.K.); hankyu29.lee@samsung.com (H.L.); 2 Department of Electronics Engineering, Chungnam National University, Daejeon 34134, Korea

**Keywords:** neural spike, instrumentation amplifier, analog nonlinear energy operator, spike detection

## Abstract

This paper presents an ultralow power 0.6 V 116 nW neural spike acquisition integrated circuit with analog spike extraction. To reduce power consumption, an ultralow power self-biased current-balanced instrumentation amplifier (IA) is proposed. The passive RC lowpass filter in the amplifier acts as both DC servo loop and self-bias circuit. The spike detector, based on an analog nonlinear energy operator consisting of a low-voltage open-loop differentiator and an open-loop gate-bulk input multiplier, is designed to emphasize the high frequency spike components nonlinearly. To reduce the spike detection error, the adjacent spike merger is also proposed. The proposed circuit achieves a low IA current consumption of 46.4 nA at 0.6 V, noise efficiency factor (NEF) of 1.81, the bandwidth from 102 Hz to 1.94 kHz, the input referred noise of 9.37 μVrms, and overall power consumption of 116 nW at 0.6 V. The proposed circuit can be used in the ultralow power spike pulses acquisition applications, including the neurofeedback systems on peripheral nerves with low neuron density.

## 1. Introduction

Ultralow power consumption is highly required to avoid overheating surrounding tissues in many neuroprosthetic devices, as well as to operate the device for long term with limited power capacity under implanted condition [1]. In neural engineering of the central nervous system, using multi electrodes array, recording of the entire waveforms are required in order to classify the features from different neurons. However, in neurofeedback applications on peripheral nerves with low neuron density, the low supply voltage neural signal processing with only the spike features rather than dealing with the entire signal waveform can be a low power solution. The typical characteristics of the neural spikes are summarized in Table 1 [1,2,3,4,5].

The spikes are rare events in neural signal with 10–120 fires/s?therefore, it is desirable to record only the spikes in order to reduce the power consumption while preserving the important information of the neuronal activities. To record the full waveform of the neurosignals, the amplifier with the acquisition bandwidth higher than the interested neural frequency range of typical 5–10 kHz is required. However, in the case of the spike extraction with the digital pulse train form, the required acquisition bandwidth can be reduced to near 1 kHz, considering the low firing rate of 10–120 fires/s. To record the low frequency neural signals, high power and area consumptions are required to reduce the flicker noises. In the spike pulse extraction, the required noise specifications also can be relaxed because the spike pulse information can be obtained with the high frequency components. The relaxed noise and bandwidth specifications enables the ultralow power implementation of the neural spike extraction integrated circuit (IC).

Instrumentation amplifiers (IAs) are key building blocks of neural signal acquisition circuits. Specifically, the capacitively-coupled IA (CCIA) and current-balanced IA (CBIA) are widely used for low-power, and low-noise biopotential acquisition [6]. In addition, the CCIA has PVT-tolerant characteristics due to its feedback configuration, (i.e., the gain is determined by the ratio of input capacitors to feedback capacitors when the open-loop gain of the amplifier is sufficiently large). Although a recently proposed self-biased current-reuse scheme can reduce its power consumption [7], the inherent maximum bandwidth is limited by the frequency compensation for feedback stability. In contrast, the CBIA gain is mostly determined by the ratio of resistors and affected by the corner variations of the transistor parameters, including source and drain resistances. In addition, it has an open-loop operation, thus exhibiting a wider bandwidth without frequency compensation. We adopted the CBIA architecture to achieve the ultralow power consumption in the proposed IC.

The digital analysis of spike signals requires a high sampling rate of the analog-to-digital converter and presents a high-power consumption for signal processing. The typical action potentials have frequency components in the range from 100 Hz to 5 kHz, and appear up to 100 times per second. The minimum required sampling rate for these potentials is 10 kHz. Given that spikes appear sporadically in the time domain, this high-frequency sampling results in unnecessary power consumption. On the other hand, the nonlinear energy operator (NEO) is widely used to estimate the instantaneous frequency and amplitude of the signal, and it has been reported to be sensitive to signal discontinuity and superior to other energy estimators for detection in noisy signals [8,9,10]. In general, the NEO requires three analog building blocks, namely, a differentiator, a four-quadrant multiplier, and a difference amplifier. These building blocks allow to emphasize high-frequency components, such as spikes, and attenuates the low-frequency components. In addition, a threshold is usually applied on the nonlinearly emphasized output of the NEO, and the spike detection depends on threshold crossing. The threshold can be a scaled average of the NEO output using a lowpass filter (LPF). Given its simplicity, analog NEO-based spike extraction is a suitable and ultralow power solution [11].

In this paper, we propose an ultralow power neural spike acquisition IC with analog spike extraction supplied with 0.6 V. To reduce power consumption, we include a self-biased CBIA, which has a low current consumption and achieves a suitable noise efficiency factor (NEF). The passive RC LPF acts as both DC servo loop (DSL) and self-bias circuit. The ultralow power analog domain NEO composed of a low-voltage open-loop differentiator, and an open-loop gate-bulk input multiplier is designed to emphasize the high frequency spike components nonlinearly. The comparison threshold is generated by low-pass filtering the NEO output. To reduce the spike detection error by the NEO operation, the adjacent spike merger is also proposed. This paper is organized as follows: Section 2 details the design, operation principle, and circuit of the proposed ultralow power neural spike acquisition IC with analog spike extraction. Section 3 presents the fabricated IC and measurement results. Finally, conclusions and summary are described in Section 4.

## 2. Neural Spike Acquisition IC

**Figure 1:**
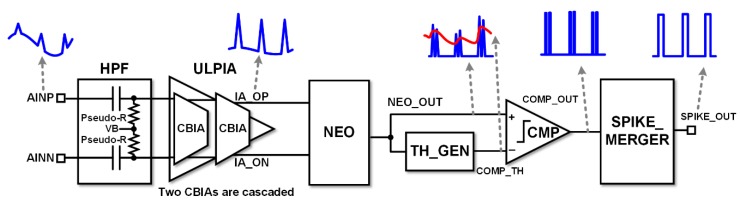
Block diagram of the proposed neural spike acquisition integrated circuit (IC).

Figure 1 shows the top-level block diagram of the proposed neural spike acquisition IC that is composed of input high pass filter (HPF), self-biased CBIA, analog NEO, comparator (CMP), and spike merger (SPIKE_MERGER) circuit. The neural input signal from electrodes, AINP and AINN, is high-pass filtered through pseudo-resistors with an adjustable corner frequency from 0.4 Hz to 115 Hz. To reduce power consumption, we designed the ultralow power IA (ULPIA) using two stage cascaded CBIAs. In the CBIA, the RC LPF acts as DSL, and also provides self-biased operation.

**Figure 2:**
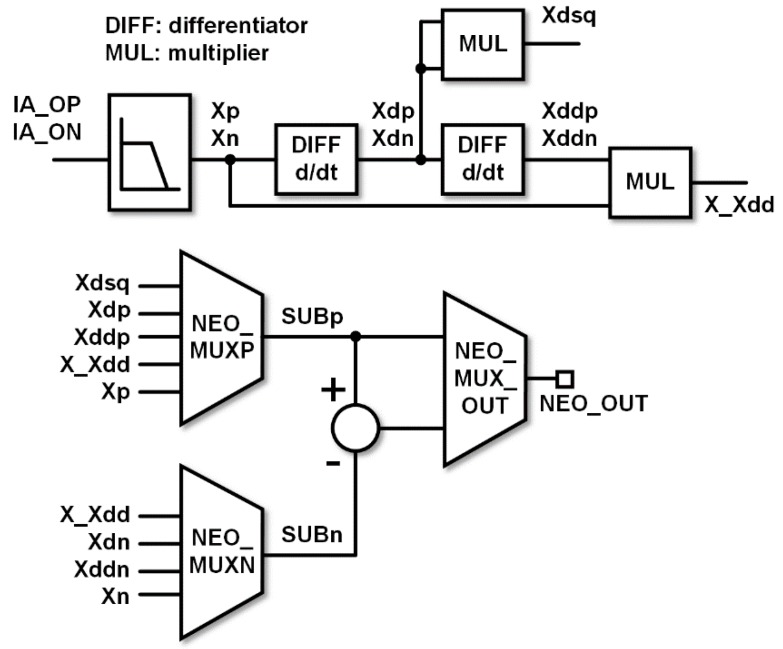
Block diagram of reconfigurable nonlinear energy operator (NEO).

The general continuous NEO is defined as

(1) $$\psi \left(x(t)\right)={\left(\frac{dx(t)}{dt}\right)}^{2}-x(t)\frac{d^{2}x(t)}{dt^{2}},$$

where *x*(*t*) is the input signal. Although the input HPF and DSL remove the low-frequency components, baseline fluctuations are still present in the output signal of the IA, between IA_OP and IA_ON. At the next stage, the analog NEO attenuates the low-frequency components and emphasizes the high-frequency spike-related components. Moreover, we designed the analog NEO to be reconfigurable into other energy operator schemes as shown in Figure 2.

The TH_GEN generates the threshold voltage for spike detection, whose level is compared with the NEO output at the comparator. The comparator output usually includes adjacent multiple pulses driven from the nonlinear operation of the NEO corresponding to one neural spike. Such multiple pulses cannot be eliminated perfectly by using a hysteresis comparator. Hysteresis can undermine the spike detection sensitivity. Therefore, we designed an adjacent spike merger circuit, which merges the adjacent spikes within a predefined time window. In the adjacent spike merger circuit, a counting clock with 6.75 kHz is used, and the merging window is programmable from 0.30 ms to 2.37 ms. Assuming the rare firing rate of typically less than 120 fires/s in neural spike activity, the merging window of the proposed spike merger circuit is shorter than the spike firing periods. Thus, the spiker merger can effectively reduce the detection error of the analog NEO.

### 2.1. Ultralow Power Self-Biased CBIA

**Figure 3:**
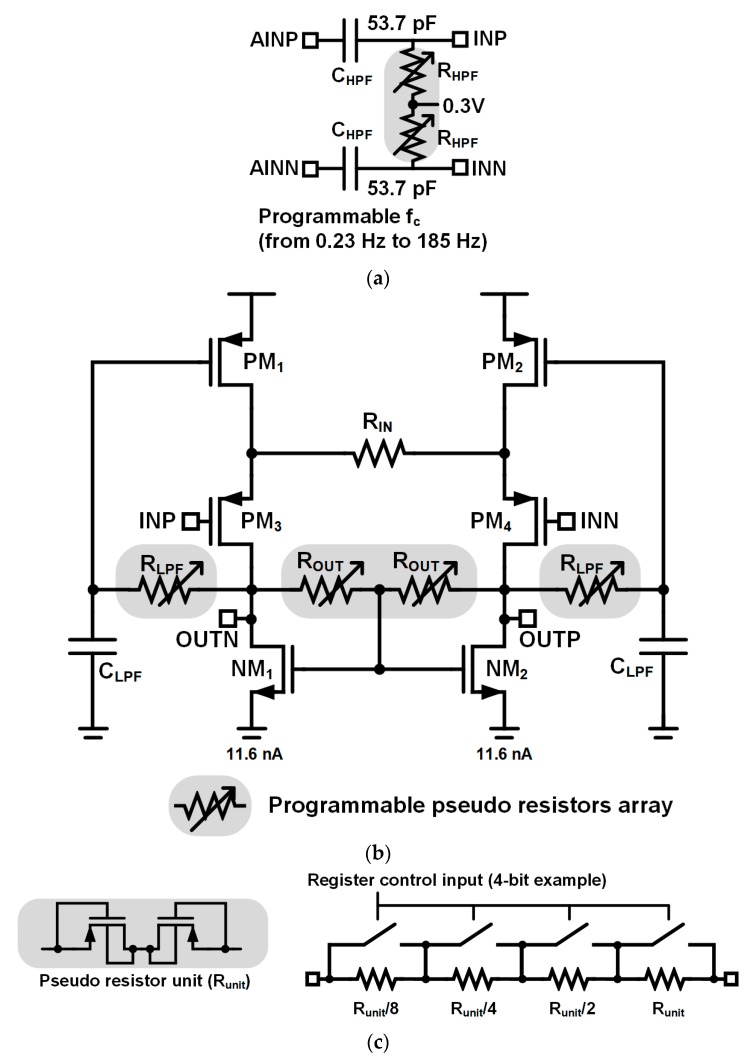
Ultralow-power self-biased current-balanced IA (CBIA): (**a**) Input passive HPF (programmable from 0.23 Hz to 185 Hz); (**b**) CBIA; (**c**) Pseudo resistors array.

The circuit diagram of proposed ultralow power self-biased CBIA is shown in Figure 3. The input signals are high-pass filtered using the input HPF, as shown in Figure 3a, and the filtered signals are amplified by the CBIA, as shown in Figure 3b. Figure 3c shows the implementation of the pseudo-resistors array. In Figure 3b, the input transistor pair, PM_3_ and PM_4_, constitutes source followers, and thus the voltage across *R*_IN_ becomes the buffered copy of the differential input voltage between INP and INN. The CBIA is designed using the medium threshold voltage (Vth) transistors, which have lower Vth than the normal Vth transistors in typical 0.18 μm process. The PM_3_ and PM_4_ have the operation points of |V_GS_| = 223 mV, |Vth| = 132 mV, |V_DS_| = 250 mV, and are operated in the saturation region. The voltage difference across *R*_IN_ results in the current difference of PM_3_ and PM_4_. Then, the differential output voltage between OUTP and OUTN is determined by the multiplication of this current difference and output resistance *R*_OUT_, which is implemented using the adjustable pseudo-resistor. The gain of the IA is proportional to the ratio between resistors *R*_IN_ and *R*_OUT_. The central node between the two pseudo-resistors *R*_OUT_ is connected to gate of n-type metal oxide semiconductor (NMOS) loads NM_1_ and NM_2_ to form a resistive common-mode feedback.

Adjustable pseudo-resistors *R*_LPF_ and metal–insulator–metal (MIM) capacitors *C*_LPF_ form the passive LPF, whose output is connected to the gates of PMOS current sources PM_1_ and PM_2_. Therefore, the low-frequency components of the output signals are negatively returned using this feedback loop, and the IA can be operated in self-bias mode. In this system, the input HPF rejects the DC components of the input signal. The negative feedback of passive LPF using R_LPF_ and C_LPF_ forms DSL, and the additional HPF characteristics can be obtained. Given that the DC gain of the passive LPF is unity, the DC rejection performance of this DSL is worse than that using active Miller integrators. However, this DSL does not consume additional power. Moreover, the gate bias voltages of PM_1_ and PM_2_ are provided by the LPF, and the IA can be operated in the self-biased mode. The DC bias current of both NM_1_ and NM_2_ is 11.6 nA, and the IA current consumption is 23.2 nA with supply voltage of 0.6 V. To achieve adequate amplification gain, two IAs are cascaded. The design values of the CBIA are summarized in Table 2.

### 2.2. Analog NEO-Based Spike Extraction

The NEO is known to outperform other spike extraction methods in conditions such as low SNR [9,10,11]. Therefore, we implemented the spike detector based on the NEO using an open-loop differentiator and a subthreshold four-quadrant multiplier, also with supply voltage of 0.6 V. The open-loop configuration of the differentiator (differentiator (DIFF) in Figure 2) and the gate-bulk input scheme of the multiplier (multiplier (MUL) in Figure 2) are based on the design in [11].

**Figure 4:**
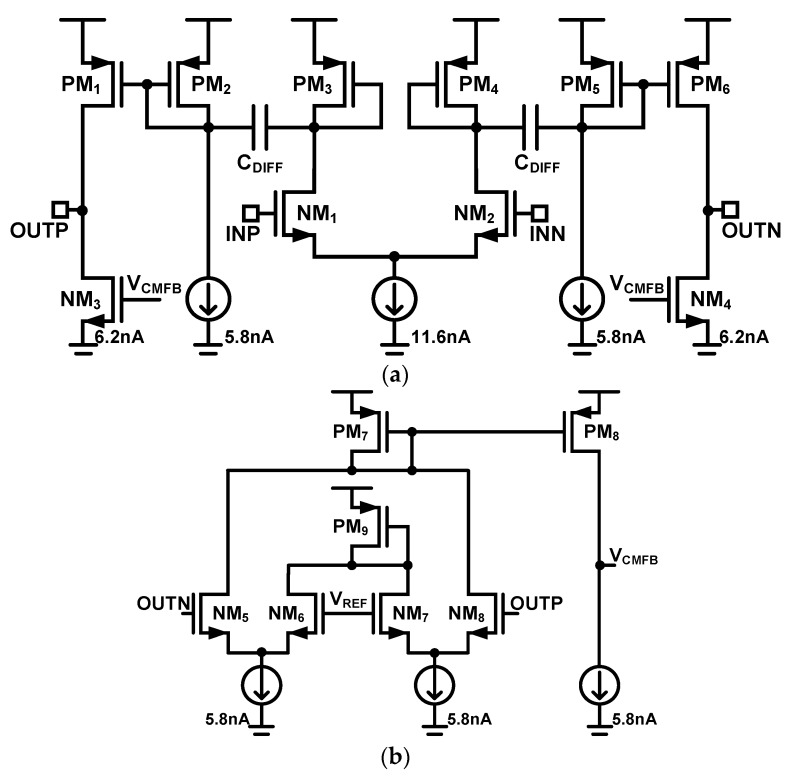
Open-loop differentiator: (**a**) Core and (**b**) common-mode feedback.

The open-loop differentiator and its common-mode feedback circuit are illustrated in Figure 4a,b, respectively. The differentiator does not require an operational amplifier and can achieve a low current consumption of 53 nA including the feedback circuit. The differentiator gain can be expressed as

(2) $$A(s)=A_{o}\frac{sC_{DIFF}}{1+sC_{DIFF}\left(\left(r_{op4}//r_{op6}\right)+\left(\frac{1}{g_{mp5}}//r_{oCS}\right)\right)},$$

where

(3) $$A_{o}=g_{mn2}g_{mp6}\left(r_{on2}//r_{op4}\right)\left(\frac{1}{g_{mp5}}//r_{oCS}\right)\left(r_{op4}//r_{op6}\right),$$

and the *r_oCS_* is the output resistance of the 5.8 nA current source. The zero of the transfer function is at the origin, and the input signal is differentiated before the dominant pole. The design values of the differentiator are summarized in Table 3.

**Figure 5:**
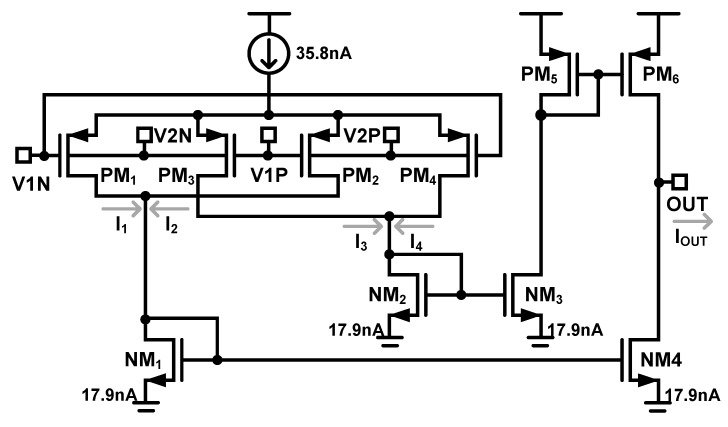
Subthreshold four-quadrant multiplier using gate and bulk input.

Figure 5 shows the design of the subthreshold four-quadrant multiplier that uses a crossed-coupled quad structure, where differential multiplication is obtained by driving the gate and bulk of the four PMOS transistors, PM_1_, PM_2_, PM_3_, and PM_4_, which operate in the subthreshold region, and whose output current can be approximated to a first-order equation given by

(4) $$I_{OUT}=(I_{3}+I_{4})-(I_{1}+I_{2})\propto V_{1}\cdot V_{2}.$$

The multiplier current consumption is 71.6 nA. The design values of the multiplier are summarized in Table 4.

**Figure 6:**
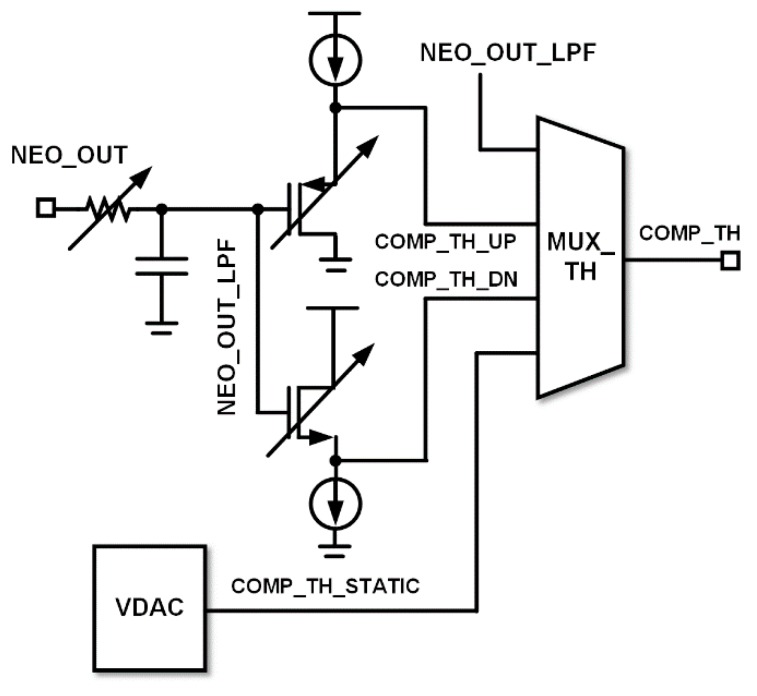
Threshold generator (TH_GEN) circuit diagram.

The threshold voltage (COMP_TH) for the comparator input is generated in the TH_GEN block as shown in Figure 6. The threshold voltage can be selected among NEO_OUT_LPF, COMP_TH_UP, COMP_TH_DN, and COMP_TH_STATIC. The static threshold (COMP_TH_STATIC) is generated from the voltage digital-to-analog converter. The low pass filtered signal (NEO_OUT_LPF) from the NEO output (NEO_OUT) is fed to the analog level shifter (source follower). In the case of positive spike detection, the low pass filtered signal (NEO_OUT_LPF) should be down-shifted to COMP_TH_DN. In the case of negative spike detection, the low pass filtered signal (NEO_OUT_LPF) should be up-shifted to COMP_TH_UP.

**Figure 7:**
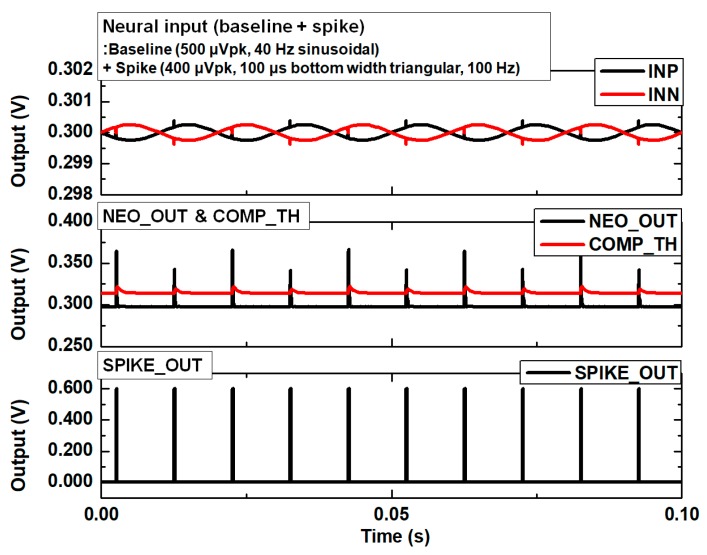
Transient noise simulation results of spike detection.

The simulation results of the spike detection with typical neural input signals are shown in Figure 7. The neural inputs, between INP and INN, include the baseline components and the spike components. The baseline components are sinusoidal with the frequency of 40 Hz and the amplitude of 500 μVpk. The spike pulse components are triangular with the bottom pulse width of 100 μs, peak amplitude of 400 μV, and firing rate of 100 Hz. In the simulation, the full spike detection chain path from input HPF, ULPIA, NEO, and the TH_GEN is included. The transient noise simulation is performed with the noise bandwidth from 0.1 Hz to 100 kHz. The simulation results show that the typical neural spikes can be detected properly under baseline fluctuation.

**Figure 8:**
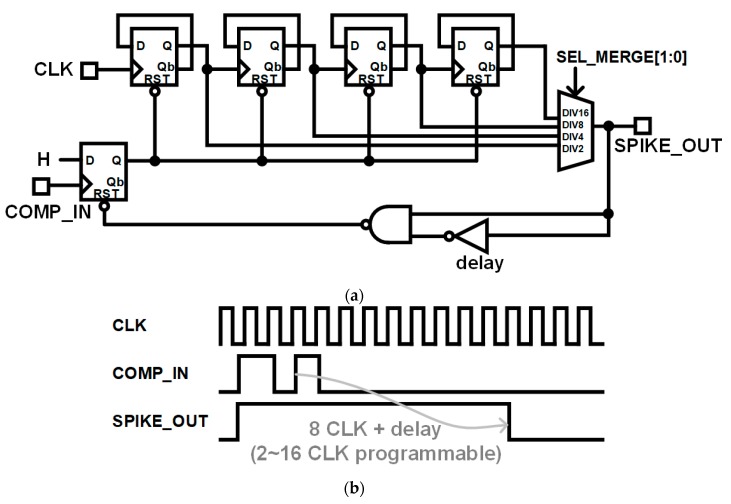
Adjacent spike merger circuit: (**a**) Circuit diagram and (**b**) timing diagram illustrating spike merging.

The NEO output usually includes adjacent multiple spikes responsive to one neural spike input. In the proposed design, we remove such glitches by merging adjacent spikes within very short intervals that contain them. Figure 8a,b show the circuit diagram and typical timing diagram of the adjacent spike merger.

In the adjacent spike merger circuit, a counting clock (CLK) with 6.75 kHz is used, and the spike merging window can be programmable from 2 CLK to 16 CLK (0.30 ms to 2.37 ms). Assuming the rare firing rate of typically less than 120 fires/s in neural spike activity, the merging window of the proposed spike merger circuit is shorter than the spike firing periods. Thus, the spike merger can effectively reduce the detection error of the analog NEO. As shown in Figure 8b, the adjacent spike merger circuit maintains signal SPIKE_OUT at the high level during merging window length after the rising edge of comparator input COMP_IN. The adjacent spikes within typical 8 CLK periods (1.19 ms) are merged to eliminate glitches.

## 3. Measurement Results

**Figure 9:**
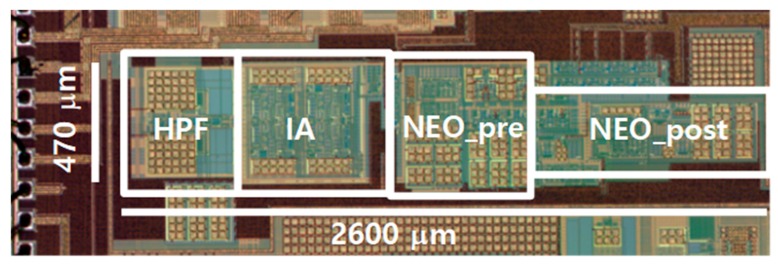
Die photo of the proposed neural spike acquisition IC.

The die photo of the proposed neural spike acquisition IC is shown in Figure 9. We fabricated the IC using the standard 0.18 μm complementary metal oxide semiconductor (CMOS) process, obtaining a chip of 470 μm × 2600 μm. The power breakdown of the IC is shown in Table 5. The supply current of the ULPIA using the 2-stage CBIA is 46.4 nA, and the total supply current of the IC is 193.3 nA with 0.6 V power supply.

**Figure 10:**
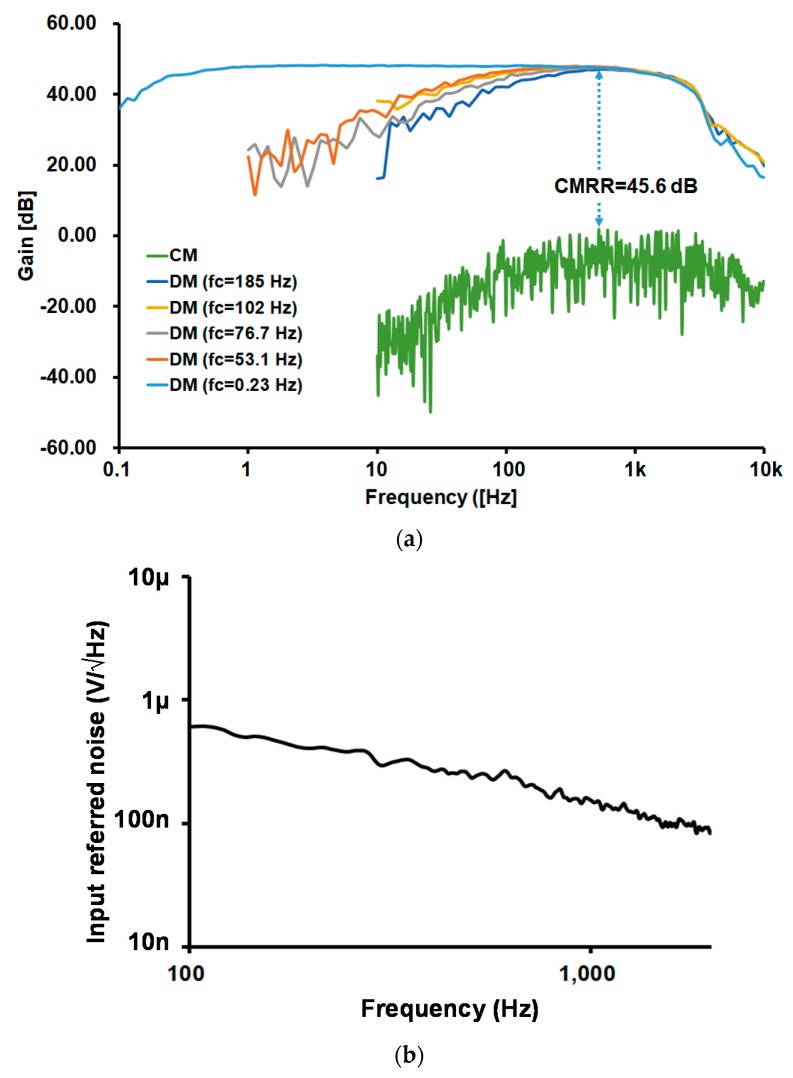
Instrumentation amplifier (IA) measurement results: (**a**) Transfer function with adjustable corner frequency and (**b**) input referred noise.

The measured transfer function and the input referred noise of the IA are shown in Figure 10a,b, respectively. The adjustable high-pass corner frequency from 0.23 Hz and 185 Hz exhibits a default gain of 48.2 dB, with a lowpass corner frequency of 1461 Hz. The input referred noise is 9.37 μVrms from 102 Hz to 1941 Hz. The current consumption of the IA is 46.4 nA at 0.6 V, and the NEF is 1.81.

**Figure 11:**
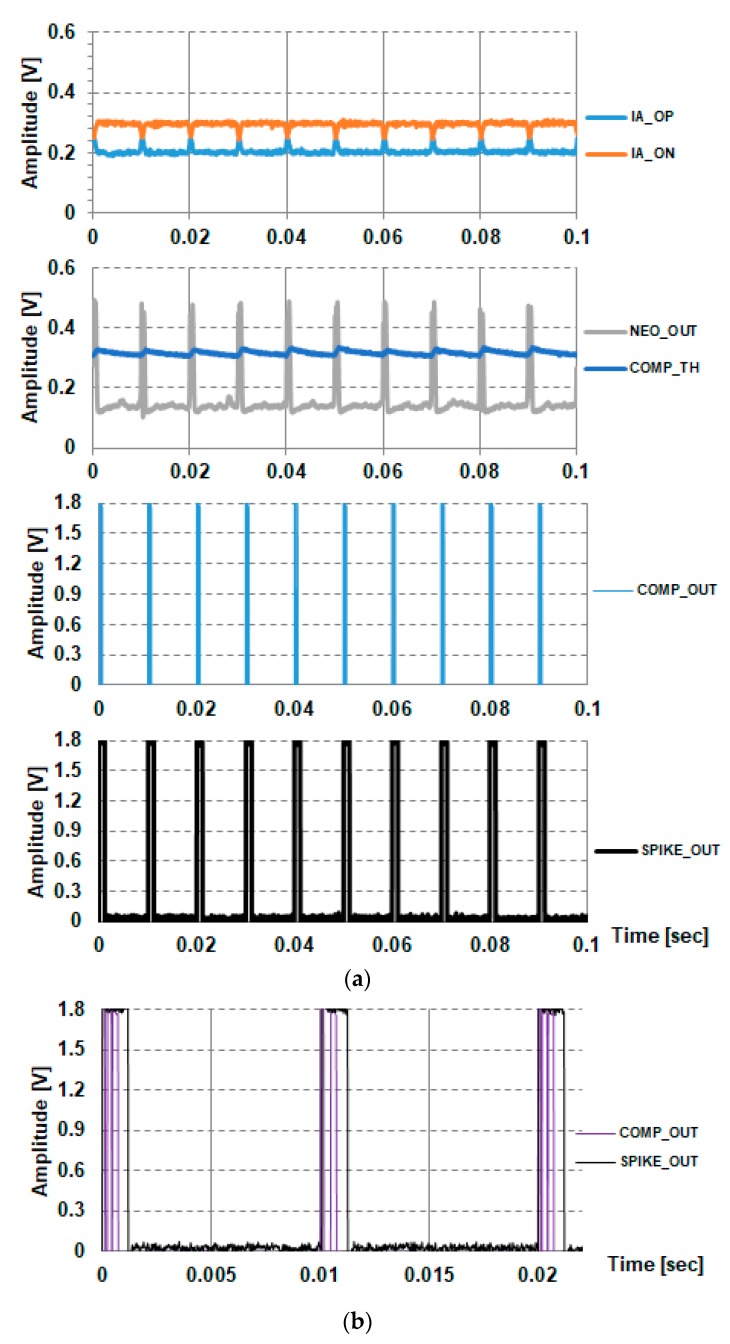
Processing of 400 μV and 100 Hz spikes: (**a**) Spike extraction and (**b**) glitch removal.

Figure 11a shows the spike detection results with an input spike amplitude of 400 μV_pk_ and intervals at 100 Hz. The spikes are properly detected, and the successful operation of the spike merger circuit is illustrated in Figure 10b. The digital signals of COMP_OUT and SPIKE_OUT are level-shifted from 0.6 V to 1.8 V for the purpose of monitoring. The typical merging window of the spike merger circuit is 1.19 ms (=840 Hz), and the high-pass corner frequency of the IA is 1461 Hz. Therefore, the maximum detectable firing rate of the input spike is limited to 840 Hz by the spike merger circuit. Because the maximum detectable firing rate is higher than the typical firing rate 120 fires/s in neural spike activity, the typical spikes can be detected properly.

Table 6 shows a performance comparison of the proposed IC to previous developments [1,7,12,13,14,15,16]. The NEF is commonly used to evaluate the efficiency in the noise-power tradeoff, and is defined as [7]

(5) $$NEF=V_{ni,rms}\sqrt{\frac{2I_{total}}{\pi \cdot U_{T}\cdot 4kT\cdot BW}}.$$

The IC shows the ultralow power consumption and good NEF and NEF∙VDD^2^. To achieve the ultralow power consumption, the input referred noise and the highpass corner frequency are increased, and the low lowpass corner frequency is decreased. In the point of view of the full waveform neural recording, the noise and bandwidth performances of the IC are not sufficient. In the point of the spike extraction applications, this IC achieves the ultralow power spike pulses acquisition with sacrificing the noise and bandwidth performance.

## 4. Conclusions

This paper presents an ultralow power neural spike acquisition IC with analog spike extraction. We fabricated the IC using the standard 0.18 μm CMOS process, obtaining a circuit of 470 μm × 2600 μm. The passive RC LPF in the CBIA acts as both DSL and self-bias. The ULPIA is implemented using the cascaded two self-biased CBIA. The ULPIA has a low current consumption of 46.4 nA at 0.6 V, NEF of 1.81, the bandwidth from 102 Hz to 1.94 kHz, and the input referred noise of 9.37 μVrms. In addition, we integrated a spike detector based on an analog NEO using a low-voltage open-loop differentiator and a gate-bulk input multiplier, and removed erroneous glitches using the adjacent spike removal circuit. Overall, the proposed IC achieves the ultralow power spike detection performance.

## Figures and Tables

**Table 1:** Typical characteristics of neural spikes.

	Interested Frequency Band	Typical Amplitude	Typical Firing Rate	Remark
ASSCC 2014 [1]	300 Hz–10 kHz	a few tens to several hundreds μV	10–100 fires/s	
TBCAS 2013 [2]	300 Hz–10 kHz	10 μV–1 mV	-	
EMBC 2013 [3]	300 Hz–5 kHz	50 μV–500 μV	-	
TNSRE 2009 [4]	100 Hz–10 kHz	50 μV–500 μV	10–120 fires/s	Duration of a few ms
JSSC 2007 [5]	300 Hz–5 kHz	50 μV–500 μV	10–100 fires/s	Duration of 250 μs

**Table 2:** Design values of CBIA.

Devices	Value
PM_1_, PM_2_	W/L = 8 μ/10 μ
PM_3_, PM_4_	W/L = 30 μ/5 μ
NM_1_, NM_2_	W/L = 0.5 μ/30 μ
*R*_IN_	35 kΩ
*R*_OUT_ (pseudo res.)	4-bit binary-weighted (LSB unit: W/L = 4 μ/1 μ, approx. 20 MOhm)
*R*_LPF_ (pseudo res.)	2-bit binary-weighted (LSB unit: W/L = 0.5 μ/10 μ, approx. 900 MOhm)
C_LPF_	17.9 pF

**Table 3:** Design values of differentiator.

Devices	Value
PM_1_, PM_2_, PM_5_, PM_6_	W/L = 0.5 μ/10 μ
PM_3_, PM_4_	W/L = 0.5 μ/30 μ
NM_1_, NM_2_	W/L = 1 μ/10 μ
NM_3_, NM_4_	W/L = 2 μ/30 μ
C_DIFF_	8.95 pF
PM_7_, PM_8_	W/L = 1 μ/10 μ
PM_9_	W/L = 5 μ/10 μ
NM_5_, NM_6,_ NM_7_, NM_8_	W/L = 2 μ/5 μ

**Table 4:** Design values of multiplier.

Devices	Value
PM_1_, PM_2_, PM_3_, PM_4_	W/L = 5 μ/10 μ
PM_5_, PM_6_	W/L = 1 μ/10 μ
NM_1_, NM_2,_ NM_3_, NM_4_	W/L = 0.5 μ/30 μ

**Table 5:** Power consumption of IC.

Sub-Block	Current (nA)
ULPIA using 2-stage CBIA	23.2 × 2 = 46.4
Differentiator	53.0
Multiplier	71.6
Threshold generator	7.2
Comparator	9.8
etc	5.3
Total	193.3

**Table 6:** Performance comparison.

	TBCAS 2009 [12]	TBCAS 2012 [13]	EL 2012 [14]	TBCAS 2014 [7]	ASSCC 2014 [1]	TCAS-II 2016 [15]	TBCAS 2017 [16]	This Work
Process (μm)	0.18	0.13	0.18	0.35	0.18	0.18	0.18	0.18
Supply voltage (V)	1.8	1.2	1	2.5	0.5	1.25	1.8	0.6
IA power (μW)	8.6	1.92	0.8	0.0825	0.7	2.125	9	0.0278
Total power/channel (μW)	23.6	2.6			1.25		56	0.116
Highpass corner freq. (Hz)	100	11.5–167	0.2–430	0.005–200	1	0.5	0.3	102 (programmable, 0.23/53.1/76.7/102/185)
Lowpass corner freq. (Hz)	9.2 k	4.8–9.8 k	5.8 k	0.1/1/10 k	6.8k	10k	7k	1.94 k
IRN (μVrms)	5.4 (100–9.2 k)	3.8 (1–100 k)	5.71	2.8 (0.05–100 Hz)	5.4 (1–12 k)	4.5 (0.5–10 k)	4.57 (0.3–7 k)	9.37 (for 102 Hz–1.94 kHz)
NEF	4.9	2.16	2.58	1.96	2.99	2.26	4.77	1.81
NEF∙VDD^2^	44.05	5.60	2.58	12.25	4.46	3.53	15.45	0.65
Spike detection	digital	analog	No	No	analog	No	analog	analog (+spike merging)

BW: Bandwidth, IRN: Input referred noise, VDD: Supply voltage, NEF: Noise efficiency factor.
